# A CD300c-Fc Fusion Protein Inhibits T Cell Immunity

**DOI:** 10.3389/fimmu.2018.02657

**Published:** 2018-11-15

**Authors:** Cheng Cui, Min Su, Yujun Lin, Laijun Lai

**Affiliations:** ^1^Department of Allied Health Sciences, University of Connecticut, Storrs, CT, United States; ^2^Department of Physiology, College of Basic Medical Science, China Medical University, Shenyang, China; ^3^University of Connecticut Stem Cell Institute, University of Connecticut, Storrs, CT, United States

**Keywords:** T cell co-inhibitory molecule, B7 family, T cell proliferation, T cell activation, GVHD

## Abstract

T cell responses are fine-tuned by co-stimulatory and co-inhibitory molecules. Among the T cell regulators, the B7 family members are of central importance. The recent success in targeting the B7 family molecules for the treatment of immune-related diseases has attracted intense interest in identifying additional B7-related molecules. In this study, we describe CD300c as a novel T cell co-inhibitory molecule that shares significant sequence homology with existing B7 family members. CD300c protein is expressed on professional antigen-presenting cells (APC), including B cells, monocytes, macrophages, and dendritic cells (DCs). The putative CD300c counter-receptor is expressed on CD4 and CD8 T cells, and the expression levels are upregulated upon activation. Soluble human and mouse CD300c-Fc fusion proteins significantly inhibit the proliferation, activation, and cytokine production by CD4 and CD8 T cells *in vitro*. Administration of CD300c-Fc protein attenuates graft-vs.-host disease (GVHD) in mice. Our results suggest that therapeutic interaction with the CD300c inhibitory pathway may represent a new strategy to modulate T cell-mediated immunity for the treatment of GVHD and autoimmune disease.

## Introduction

T cell immune responses are tightly controlled by co-stimulatory and co-inhibitory molecules. The co-stimulatory molecules contribute to the development of immune responses against cancers and foreign pathogens, while the co-inhibitory molecules are critical for peripheral tolerance to avoid autoimmunity, GVHD and transplant rejection.

A number of T cell co-stimulatory and co-inhibitory ligands and receptors have been identified. Among them, B7 family members are of central importance. The B7 family ligands include B7-1 (1), B7-2 (2), PD-L1 (B7-H1) (3, 4), PD-L2 (B7-DC) (5, 6), inducible T cell co-stimulator ligand (ICOSL) (also known as, B7-H2, B7h, B7RP-1, GL50, LICOS) (7–10), B7-H3 (11), B7-H4 (B7x, B7S1) (12–14), B7-H5 (HHLA2) (15, 16), and B7-H6 (17), etc. The importance of these molecules has been highlighted by the FDA approval of several drugs for the treatment of cancer and autoimmune disease by targeting the ligands or their receptors. For example, recombinant fusion protein CTLA-4-Fc (Abatacept or Balatacept), an inhibitory receptor for B7-1 and B7-2, has been used in the treatment of rheumatoid arthritis and kidney transplantation rejection. In contrast, antibodies against PD-L1/PD-1 or CTLA-4, such as Pembrolizumab (Keytruda), Nivolumab (Opdivo), Atezolizumab (Tecentriq), Avelumab (Bavencio), Durvalumab (Imfinzi), Ipilimumab (Yervoy), can significantly enhance antitumor immunity and the survival of cancer patients.

The unprecedented success by targeting the ligands or receptors of the B7 family members for the treatment of cancer and autoimmune disease has attracted considerable interest in identifying additional T cell regulators. In this study, we identify CD300c as a novel T cell inhibitory molecule. Human CD300c (hCD300c), also known as CMRF-35A or CMRF-35, was originally isolated from human leukocytes (18–20). It has been reported that CD300c has effects on NK cells, monocytes, macrophages, B cells, DCs and basophils (21–30). CD300c can bind to plasma membrane lipids phosphatidylethanolamine (PE) and phosphatidylserine (PS) (23, 30, 31). However, the functional significance of CD300c on T cells has not been characterized.

We show here that hCD300c is homologous to known B7 family members in amino acid sequence. CD300c is expressed on APCs, and its putative counter-receptor is expressed on CD4 and CD8 T cells. Both soluble human and mouse CD300c-IgG2a Fc (CD300c-Ig) fusion proteins significantly inhibit the proliferation and activation, and cytokine production of CD4 and CD8 T cells *in vitro*. Administration of hCD300c-Ig protein ameliorates GVHD in mice. Therefore, this unique T cell inhibitory pathway may provide a new strategy to modulate T cell-mediated immunity to treat immune-related diseases.

## Materials and methods

### Bioinformatics analysis of CD300c

Sequence alignments of CD300c and known B7 family members, and CD300c orthologous proteins were analyzed via the Clustal W program in MacVector 16.0.5 (MacVector, Inc.). The leader peptide, transmembrane, and Ig-like domain were predicted with SignalP 4.0 (http://www.cbs.dtu.dk/services/SignalP), TMHMM server version 2.0 (http://www.cbs.dtu.dk/services/TMHMM/), and InterPro (https://www.ebi.ac.uk/interpro).

### Cloning and purification of hCD300c and mCD300c2

The extracellular domains of hCD300c (aa29-183) and mCD300c2 (aa22-193) were cloned and fused into a pCMV6-AC-FC-S expression vector containing the constant region of mouse IgG2a (ORIGENE). The vectors were transfected into HEK293F cells. The fusion proteins were purified for supernatant using Protein G Sepharose 4 Fast Flow according to the manufacturer's instructions (GE Healthcare). Purified proteins were verified by SDS-PAGE, Coomassie Staining and Western blot. Protein was quantified using the Pierce™ BCA Protein Assay Kit (Pierce, Rockford, IL). Control Ig (recombinant mouse IgG2a Fc protein) was purchased from BXCell (West Lebanon, NH).

### SDS-PAGE and western blot

Purified CD300c-Ig was loaded on a 12% SDS-PAGE, and stained with Coomassie blue or transferred to a polyvinylidene fluoride membrane. The protein containing membrane was incubated with HRP conjugated anti-mouse IgG2 antibody, or anti-hCD300c antibody (Novus Biologicals, Littleton, CO) followed by HRP conjugated second antibody, and then developed with Super Signal® West Pico chemiluminescent Substrate (Thermo Scientific).

### Flow cytometry analysis

Single cell suspensions of organs were stained with the fluorochrome-conjugated antibodies protein as described (32–35). For intracellular staining, the cells were first permeabilized with a BD Cytofix/Cytoperm solution for 20 min at 4°C. Direct or indirect staining of fluorochrome-conjugated antibodies included: CD4, CD8, CD19, B220, CD11c, CD11b, F4/80, H2^b^, Annexin V, Ki67, CD44, CD62L, CD69, CTLA-4, CD28, PD-1, BTLA, and ICOS and mCD300c2 (BioLegend or BD Biosciences, San Jose, CA, San Diego, CA). mCD300c2-Ig and hCD300c-Ig were biotinylated with sulfo-NHS-LC-Biotin (Pierce). The samples were analyzed on a FACSCalibur or LSRFortessa X-20 Cell Analyzer (BD Biosciences). Data analysis was done using FlowJo software (Ashland, OR).

### Limulus amebocyte lysate (LAL) assay

The endotoxin level in the purified proteins was determined by the endpoint chromogenic LAL test according to the manufacturer's instructions (Lonza, Walkersville, MD) (36).

### *In vitro* T cell proliferation assays

Normal human peripheral blood CD3^+^ Pan T Cells that were negatively isolated from mononuclear cells using an indirect immunomagnetic Pan-T labeling system were purchased from ALLCELLS, LLC (Alameda, CA). Murine CD3^+^ T cells were purified from C57BL/6 mice by an immunomagnetic system (Miltenyi, Auburn, CA), and the purity of the cells was usually >95%. T cells were stimulated with anti-CD3 and/or anti-CD28 antibodies (Biolegend) in the presence of CD300c-Ig or control Ig. Proliferative response was assessed by pulsing the culture with 1 μCi of [^3^H] thymidine (PerkinElmer, Inc., Downers Grove, IL) 12 h before harvest. Incorporation of [^3^H] thymidine was measured by liquid scintillation spectroscopy (PerkinElmer, Inc.). For carboxyfluorescein diacetate succinimidyl ester (CFSE) assay, splenocytes were labeled with CFSE (ThermoFisher Scientific), and stimulated with anti-CD3 in the presence of CD300c-Ig or control Ig. The cells were analyzed by flow cytometry.

### Mice

Four-week-old female C57BL/6 and BALB/c mice were purchased from Jackson Laboratory. The mice were used in accordance with a protocol approved by the Institutional Animal Care and Use Committee of the University of Connecticut.

### GVHD model

BALB/c recipients received 900 cGy total body irradiation from a 137Cs source (Gammator-50 Gamma Irradiator; Radiation Machinery Corporation, Parsippany, NJ). Two to four hours later, the mice were injected intravenously (i.v.) with BM and spleen cells from C57BL/6 mice. The recipients were injected i.p. with hCD300c-Ig, or control Ig. The severity of GVHD was evaluated with a clinical GVHD scoring system. In brief, GVHD recipients in coded cages were individually scored every week for five clinical parameters on a scale from 0 to 2: weight loss, posture, activity, fur texture and skin integrity. A clinical GVHD index was generated by summation of the five criteria scores (maximum index = 10).

GVHD target organs were harvested for histopathological analysis. The organs were formalin-preserved, paraffin-embedded, sectioned and hematoxylin/eosin (H&E)-stained. Assessment of tissue damage was performed based on scoring systems previously described (37). Briefly, liver GVHD was scored on the number of involved tracts and severity of liver cell necrosis; the maximum score is 10. Gut GVHD was scored on the basis of crypt apoptosis and lamina propria inflammation; the maximum score is 8. Lung GVHD was scored on the periluminal infiltrates, pneumonitis, and the severity of lung tissues involved; the maximum score is 9.

### Statistical analysis

*P*-values were based on the two-sided Student's *t*-test. A confidence level above 95% (*p* < 0.05) was determined to be significant.

## Results

### CD300c shares sequence and structural homology with the B7 family molecules

**Figure 1:**
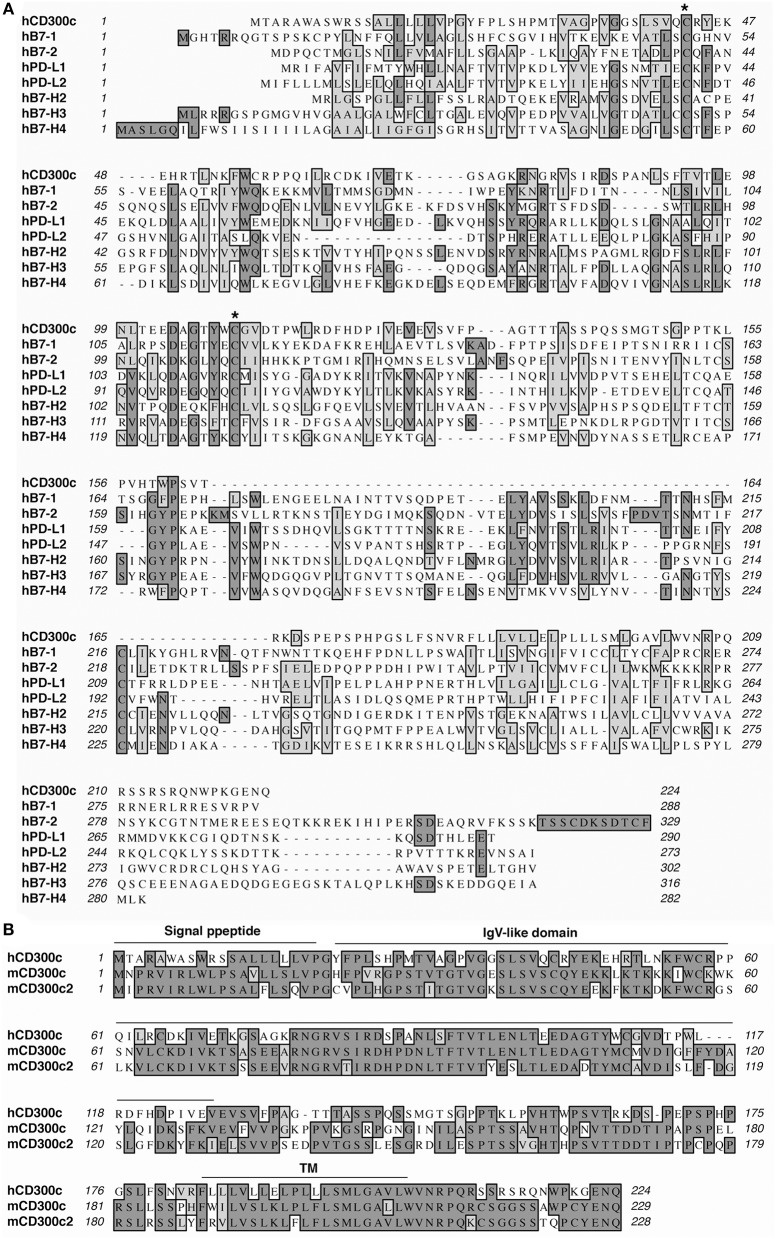
CD300c is a B7 family-related molecule. **(A)** Alignment of hCD300c with some known B7 family members. Identical amino acids are shaded black. Amino acids with strong homologies are shaded in gray. Conserved cysteine residues are labeled with an asterisk (*). **(B)** Alignment of hCD300c with mCD300c and mCD300c2. Predicted signal peptide, IgV-like, and transmembrane (TM) domains for hCD300 are marked.

Recognizing the importance of the B7 family in controlling immune responses, we performed a series of genome-wide database searches to find molecules that are homologous to known B7 family members. We discovered that hCD300c shares varying levels of amino acid identity and similarity with B7-1 (17 and 13%), B7-H2 (16 and 12%), B7-H3 (13 and 12%), B7-H4 (12 and 15%), PD-L1 (14 and 19%), and PD-L2 (13 and 15%) (Figure 1A). It has been reported that human B7-1 shares 13–21% of amino acid identity with other B7 family members (15). The levels of amino acid identity of hCD300c with the known B7 family members suggest that CD300c is a B7 family-related molecule.

It has been reported that the mouse orthologs of hCD300c are mouse CD300c (mCD300c) [also called CMRF-35-like molecule-6 (CLM-6)] and mCD300c2 [also known as leukocyte mono-Ig-like receptor 2 (LMIR2), dendritic cell-derived Ig-like receptor 1 (DIgR1), myeloid-associated Ig-like receptor II (MAIR-II), or CLM-4] (24–28). hCD300c shares 51 and 48% identity, and 6 and 8% similarity with mCD300c and mCD300c2, respectively (Figure 1B). mCD300c and mCD300c2 also share 8–10% amino acid identity and 9–14% amino acid similarity with mouse B7-1, B7-H2, B7-H3, B7-H4, PD-L1, and PD-L2 (Supplemental Figure 1). hCD300c, mCD300c, and mCD300c2 belong to the immunoglobulin (Ig) superfamily and are type I transmembrane proteins that contain an extracellular region with a single Ig-V like domain, a transmembrane segment, and a short cytoplasmic tail (Figure 1B) (18–22, 38, 39).

### hCD300c inhibits the proliferation and activation of mouse and human T cells *in vitro*

To investigate whether like the known B7 family members, CD300c protein can affect T cell function, we produced an hCD300c-Ig fusion protein by cloning the extracellular domain of the hCD300c gene into an expression vector containing the constant region of the mouse IgG2a. The expression vector was then transfected into human HEK-293 cells to produce hCD300c-Ig fusion protein that was then purified from the supernatant of the cells. A relatively high purity of hCD300c-Ig protein was obtained, as determined by Coomassie blue-stained SDS-PAGE (Supplemental Figure 2). The identity of the fusion protein was verified by Western blot using anti-IgG2a antibody or anti-hCD300c antibody (Supplemental Figure 2). The actual molecular weight (MW) of the hCD300c-Ig was higher than the predicted MW, suggesting that the recombinant protein was glycosylated. The endotoxin level was < 0.01 EU/ml of 1 μg of purified protein.

**Figure 2:**
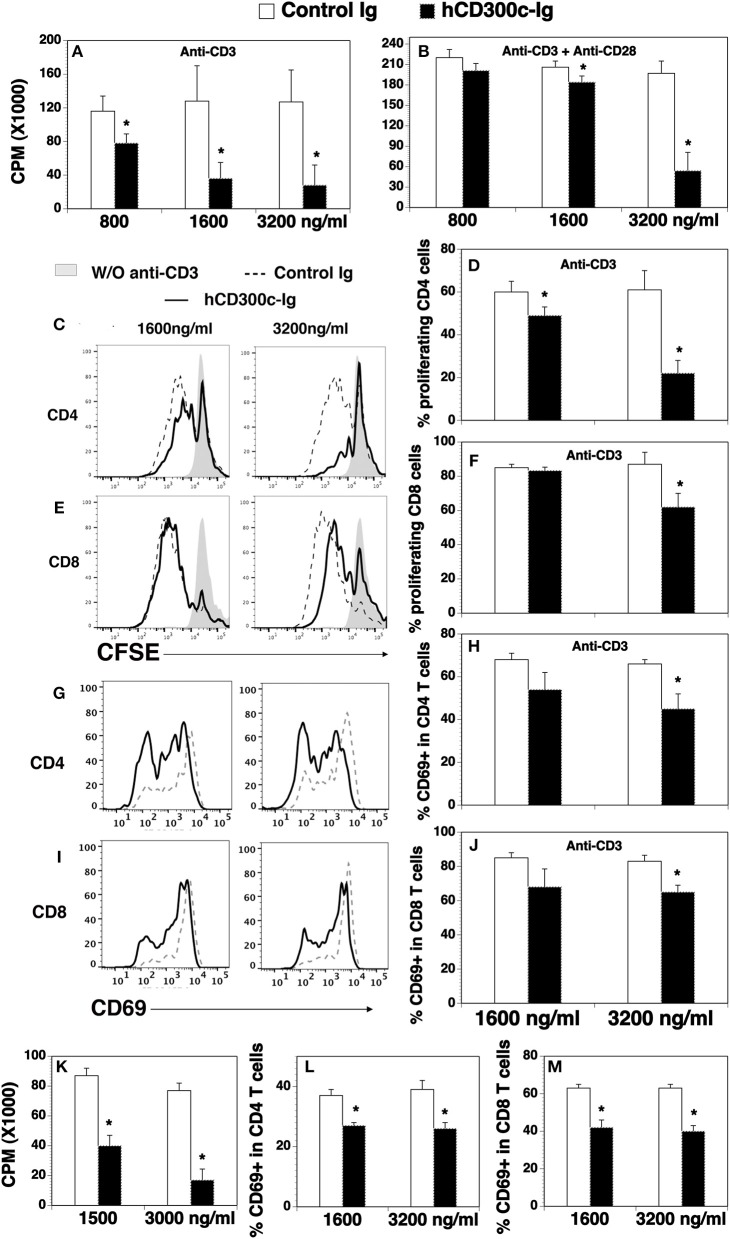
The effects of hCD300c-Ig protein on the proliferation and activation of mouse and human T cells *in vitro*. **(A,B)** T cells were isolated from the spleen of C57BL/6 mice and cultured with **(A,B)** plate-bound anti-CD3 antibody (1 μg/ml) and **(B)** anti-CD28 antibody (0.5 μg/ml) in the presence of graded doses of hCD300c-Ig protein (800, 1,600, and 3,200 ng/ml) or equimolar amounts of control Ig protein for 3 days. Cell proliferation was measured by [^3^H] thymidine incorporation. **(C–F)** Mouse splenic cells were labeled with CFSE and cultured with anti-CD3 antibody (1 μg/ml) and graded doses of hCD300c-Ig protein (1,600 and 3,200 ng/ml) or equimolar amounts of control Ig protein for 3 days. The cells were stained with anti-CD4 and CD8 antibodies, and analyzed for CFSE levels by CD4^+^ and CD8^+^ T cells. **(C,E)** Representative flow cytometric profiles and **(D,F)** statistical analysis of CFSE^lo^ proliferating CD4^+^ or CD8^+^ T cells. **(G–J)** Mouse splenic cells were cultured with anti-CD3 antibody and graded doses of hCD300c-Ig protein or control Ig protein as **(C)** for 24 h. The cells were then analyzed for CD69 expression by CD4^+^ and CD8^+^ T cells. **(G,I)** Representative flow cytometric profiles, and **(H,J)** statistical analysis of CD69^+^ cells in CD4^+^ or CD8^+^ T cells. **(K)** Purified human CD3^+^ T cells were cultured with plate-bound anti-human CD3 antibody (1 μg/ml) in the presence of graded doses of hCD300c-Ig protein (1,500 and 3,000 ng/ml) or control Ig protein for 3 days. Cell proliferation was measured by [^3^H] thymidine incorporation. **(L,M)** Purified human CD3^+^ T cells were cultured with plate-bound anti-human CD3 antibody (1 μg/ml) in the presence of graded doses of hCD300c-Ig protein (1,600 and 3,200 ng/ml) or control Ig protein for 1 days. The cells were then analyzed for human CD69 expression by CD4^+^ and CD8^+^ T cells. The data were pooled from 3 independent experiments and expressed as mean ± SD. *P < 0.05 compared with control Ig.

We then determined whether hCD300c-Ig protein affected T cell proliferation. To do this, CD3^+^ T cells were purified from splenocytes of C57BL/c mice, and cultured on plates pre-coated with anti-CD3 antibody in the presence of graded doses of hCD300-Ig (800, 1,600, and 3,200 ng/ml) for 3 days. Since the molecular weight of hCD300-Ig fusion protein is ~1.5-fold higher than that of control Ig protein, we used equimolar amounts of the control Ig as a control. T cell proliferation was measured by [^3^H] thymidine incorporation. As shown in Figure 2A, hCD300c-Ig inhibited anti-CD3-activated T cell proliferation in a dose-responsive manner, with ~32, 72, and 78% inhibition by 800, 1,600, and 3,200 ng/ml hCD300c-Ig, respectively, as compared to equimolar amounts of control Ig. We also determined whether hCD300c could inhibit anti-CD3 and anti-CD28 antibody-activated T cell proliferation. Similarly, hCD300c-Ig reduced anti-CD3 and anti-CD28-activated T cell proliferation in a dose-dependent manner, although to a lesser extent than that with anti-CD3 activation only (Figure 2B).

To confirm the effect on T cell proliferation and to determine whether hCD300c affects CD4 and/or CD8 T cells, we performed a carboxyfluorescein diacetate succinimidyl ester (CFSE) dilution assay. Murine splenocytes were labeled with CFSE, and then cultured with anti-CD3 antibody in the presence of graded doses of hCD300c-Ig or control Ig. T cell proliferation was measured by CFSE fluorescent dilution in CD4 and CD8 T cells. As shown in Figures 2C–F, hCD300c-Ig inhibited anti-CD3-activated proliferation of both CD4 and CD8 T cells in a dose-dependent manner.

We next determined whether hCD300c-Ig affects the activation of T cells *in vitro*. CD69 is an early activation marker. After splenocytes were cultured with anti-CD3 antibody and hCD300c-Ig or control Ig, the expression of the CD69 on CD4 and CD8 T cells was analyzed 24 h later. As shown in Figures 2G–J, hCD300c-Ig at the dose of 3,200 ng/ml significantly reduced the expression of CD69 on both CD4 and CD8 T cells. The results suggest that hCD300c also inhibits the activation of CD4 and CD8 T cells.

Having demonstrated that hCD300c-Ig inhibited murine T cells proliferation *in vitro*, we examined whether hCD300c-Ig affected human T cells. Purified human T cells were cultured with anti-CD3 antibody in the presence of graded doses of hCD300-Ig or control Ig, and T cell proliferation was measured by [^3^H] thymidine incorporation. Similarly, hCD300c-Ig markedly inhibited human T cell proliferation with ~53 and 77% inhibition by 1,500, and 3,000 ng/ml hCD300c-Ig, respectively (Figure 2K). Furthermore, hCD300c-Ig at both 1,600 and 3,200 ng/ml doses significantly reduced the expression of CD69 on both human CD4 and CD8 T cells (Figures 2L,M).

Taken together, our results suggest that hCD300c-Ig inhibits TCR-mediated proliferation and/or activation of both mouse and human T cells *in vitro*. hCD300c has similar inhibitory effects in both human and mouse primary T cells, suggesting that its binding partner and its conferred function on T cells may be conserved across species.

### mCD300c2 inhibits the proliferation and activation of mouse T cells *in vitro*

**Figure 3:**
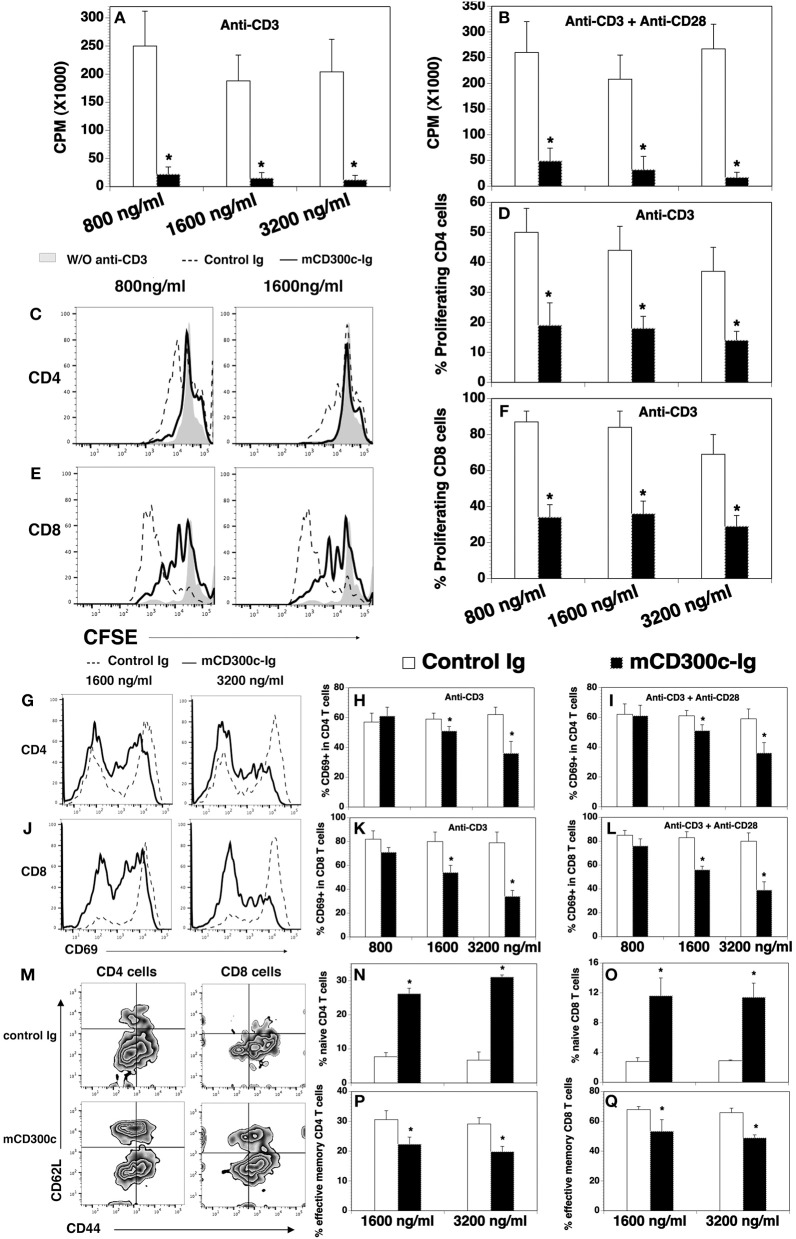
The effects of mCD300c-Ig protein on the proliferation and activation of mouse T cells *in vitro*. **(A,B)** CD3^+^ T cells were isolated from the spleen of C57BL/6 mice and cultured with **(A,B)** plate-bound anti-CD3 antibody (1 μg/ml) and **(B)** anti-CD28 antibody (0.5 μg/ml) in the presence of graded doses of mCD300c2-Ig protein (800, 1,600, and 3,200 ng/ml) or equimolar amounts of control Ig protein for 3 days. Cell proliferation was measured by [^3^H] thymidine incorporation. **(C–F)** Mouse splenic cells were labeled with CFSE and cultured with anti-CD3 antibody (1 μg/ml) and graded doses of mCD300c2-Ig protein or control Ig protein as **(A)**. The cells were then stained with anti-CD4 and CD8 antibodies and analyzed for CFSE levels by CD4^+^ and CD8^+^ T cells. **(C,E)** Representative flow cytometric analysis of CFSE distribution of CD4^+^ or CD8^+^ T cells. **(D,F)** Statistical analysis of CFSE^lo^ proliferating CD4^+^ or CD8^+^ T cells. **(G–Q)** Mouse splenic cells were cultured with **(G–Q)** anti-CD3 antibody and **(I,L)** anti-CD28 antibody in the presence of graded doses of mCD300c2-Ig or control Ig protein as **(A,B)**. The cells were analyzed for the expression of **(G–L)** CD69 24 h later, **(M–Q)** CD44, and CD62L 72 h later. **(G,J,M)** Representative flow cytometric profiles, and **(H,I,K,L,N–Q)** statistical analyses of the percentages of CD69^+^, CD44^low^CD62L^hi^ naïve, and CD44^hi^CD62L^low^ effective memory CD4 and CD8 T cells. The data were pooled from 3 independent experiments and expressed as mean ± SD. *P < 0.05 compared with control Ig.

We also produced a mCD300c2-Ig protein by fusing the extracellular domain of mCD300c2 to the mouse IgG2a constant region, and analyzed the effects of purified mCD300c2-Ig fusion protein on mouse T cell proliferation and activation *in vitro*. We found that mCD300c2-Ig markedly inhibited anti-CD3-induced T cell proliferation, with more than 90% inhibition by 800, 1,600, or 3,200 ng/ml of mCD300c2-Ig (Figure 3A). mCD300c2-Ig also inhibited anti-CD3 and anti-CD28 antibody-induced T cell proliferation, with ~81, 85, and 94% inhibition by 800, 1,600, and 3,200 ng/ml hCD300c-Ig, respectively (Figure 3B). CFSE dilution assay confirmed that mCD300c2-Ig inhibited anti-CD3-induced proliferation of both CD4^+^ and CD8^+^ T cells (Figures 3C–F).

Like hCD300c-Ig, mCD300c2-Ig significantly reduced the expression of CD69 on both CD4^+^ and CD8^+^ T cells induced by either anti-CD3 antibody, or anti-CD3 plus anti-CD28 antibodies, and the reduction was also in dose-dependent manner (Figures 3G–L). To further confirm that mCD300c2-Ig inhibits T cell activation, we analyzed the expression of CD44 and CD62L by CD4^+^ and CD8^+^ T cells. It has been reported that naïve T cells are CD44^low^CD62L^hi^, while effective memory T cells are CD44^hi^CD62L^low^. As shown in Figures 3M–Q, mCD300c2-Ig significantly increased the percentages of CD44^low^CD62L^hi^ naïve cells in anti-CD3-activated CD4^+^ and CD8^+^ T cells, but decreased the percentages of CD44^hi^CD62L^low^ effective memory T cells. The results further suggest that mCD300c2-Ig inhibits the activation of CD4^+^ and CD8^+^ T cells.

Collectively, our results suggest that both hCD300c-Ig and mCD300c2-Ig inhibits TCR-mediated proliferation and activation of both CD4 and CD8 T cells *in vitro*, providing further evidence that CD300c is a novel B7 family-related molecule with T cell co-inhibitory properties.

### mCD300c2 inhibits cytokine production from T cells

**Figure 4:**
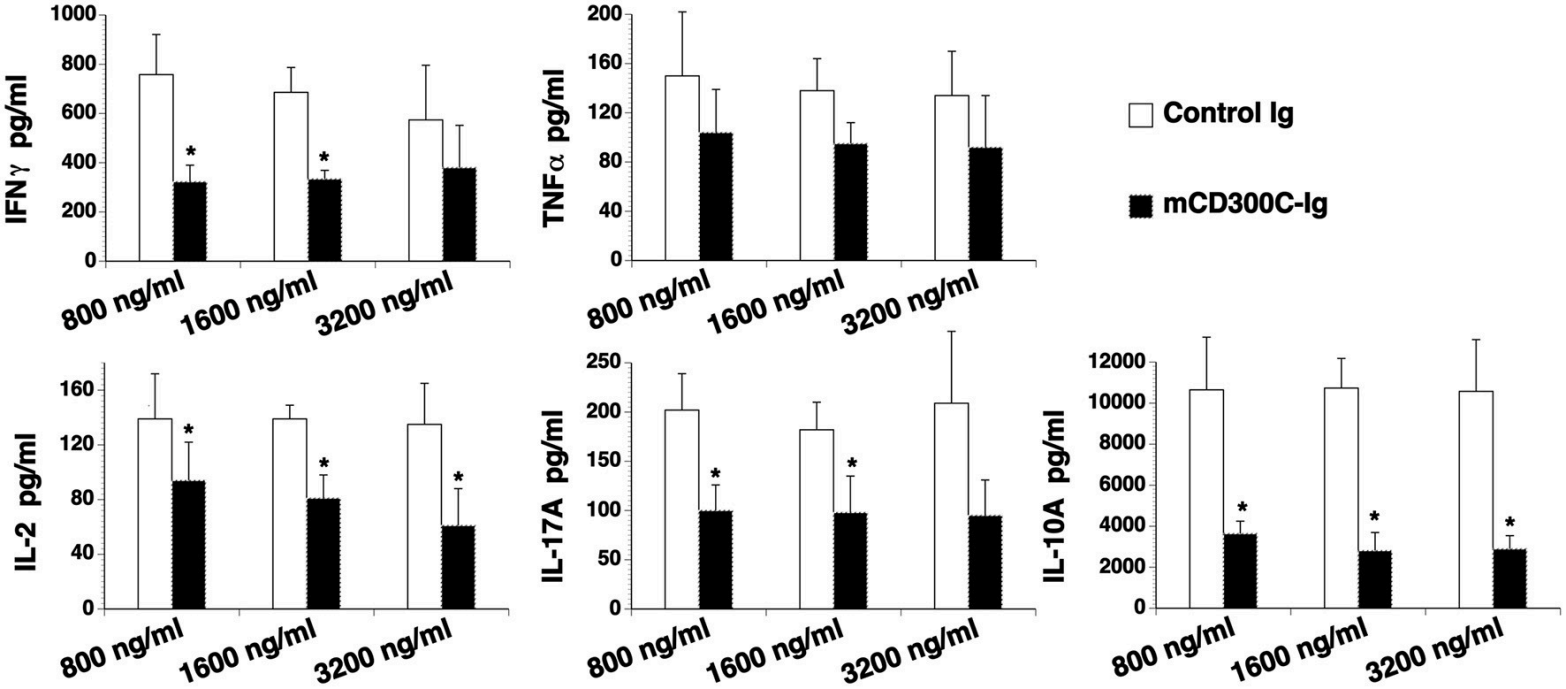
mCD300c2 suppresses cytokine production from T cells. Purified murine T cells were cultured with plate-bound anti-CD3 antibody (1 μg/ml) in the presence of graded doses of mCD300c2-Ig protein or control Ig protein for 3 days as Figure 3A. The levels of IFNγ, TNFα, IL-2, IL-17A, and IL-10 in the supernatant were measured by ELISA kits. The data were pooled from 3 independent experiments and expressed as mean ± SD. *P < 0.05 compared with control Ig.

We then determined the effect of mCD300c2 on cytokine production from T cells *in vitro*. CD3^+^ T cells were purified from the spleens of C57BL/6 mice and stimulated with anti-CD3 antibody in the presence of graded doses of mCD300c2-Ig or control Ig protein for 3 days. The contents of cytokines in the supernatants were measured by ELISA. As shown in Figure 4, mCD300c2-Ig inhibited the production of IFNγ, IL-2, IL-17A, and IL-10, but not TNFα. The results suggest that mCD300c2-Ig suppresses certain Th1/Th2/Th17 cytokine production by T cells induced by TCR signaling.

### The expression pattern of CD300c on murine immune cells

**Figure 5:**
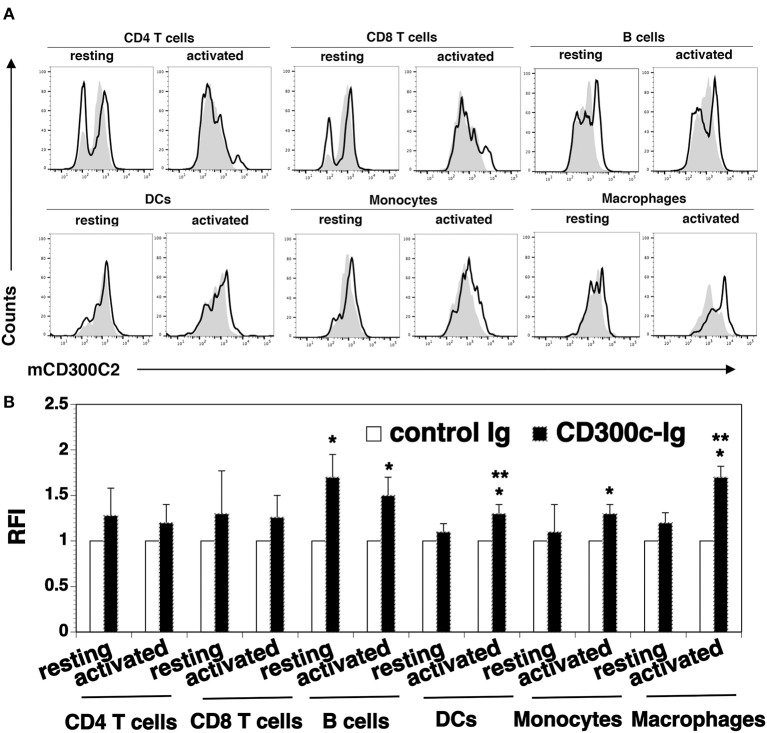
Analysis of the expression of mCD300c2 on immune cells. Splenocytes from C57BL/6 mice were freshly harvested and used for resting immune cells. To initiate T cell activation, splenocytes were incubated with anti-CD3 (1 μg/ml) and anti-CD28 (0.5 μg/ml) antibodies for 3 days. For activated B cells, DCs, monocytes and macrophages, splenocytes were incubated with LPS (10 μg/ml) for 3 days. The resting and activated immune cells were stained with anti-mCD300c2 antibody (Ab) (open histograms) or isotype Ab (shaded histograms), as well as, anti-CD4, CD8, B220, CD11c, CD11b, or F4/80 Ab to identify immune cells. **(A)** Representative flow cytometric profiles and **(B)** statistical analysis showing the expression of mCD300c2 on resting and activated immune cells. **(B)** The data were pooled from 3 independent experiments and presented as relative fluorescence intensity (RFI) for the expression of mCD300c2 on activated cells vs. resting cells. *P < 0.05 compared with isotype Ab. **P < 0.05 compared with resting cells.

We analyzed cell surface expression of mCD300c2 protein on murine immune cells by flow cytometry using a monoclonal antibody against mCD300c2 (clone TX52). Although the manufacture's manual indicates that this antibody recognizes mCD300c and mCD300d, this antibody was generated by using mouse MAIR-II (mCD300c2)-transfected cells as an immunogen. As indicated in the Discussion Section, the nomenclature for mCD300c is still confusing and MAIR-II is also known as mCD300c2/CD300d. Therefore, we believe that this antibody reacts to mCD300c2 protein. We found that resting splenic CD4^+^ and CD8^+^ T cells scarcely expressed mCD300c2 protein (Figure 5). After activation by anti-CD3 and anti-CD28 antibodies, a small percentage of activated CD4^+^ and CD8^+^ T cells expressed mCD300c2. We then examined the expression of mCD300c2 protein on other immune cells, and found that resting and activated B220^+^ B cells, CD11b^+^ monocytes and F4/80^+^ macrophages expressed various levels of mCD300c2 (Figure 5). The expression level of mCD300c2 on macrophages was upregulated upon activation. In addition, although CD300c protein was scarcely expressed on resting CD11c^+^ DCs, it was induced upon activation by LPS (Figure 5). These results suggest that CD300c is expressed on a variety of APCs.

### The expression of the putative CD300c counter-receptor

**Figure 6:**
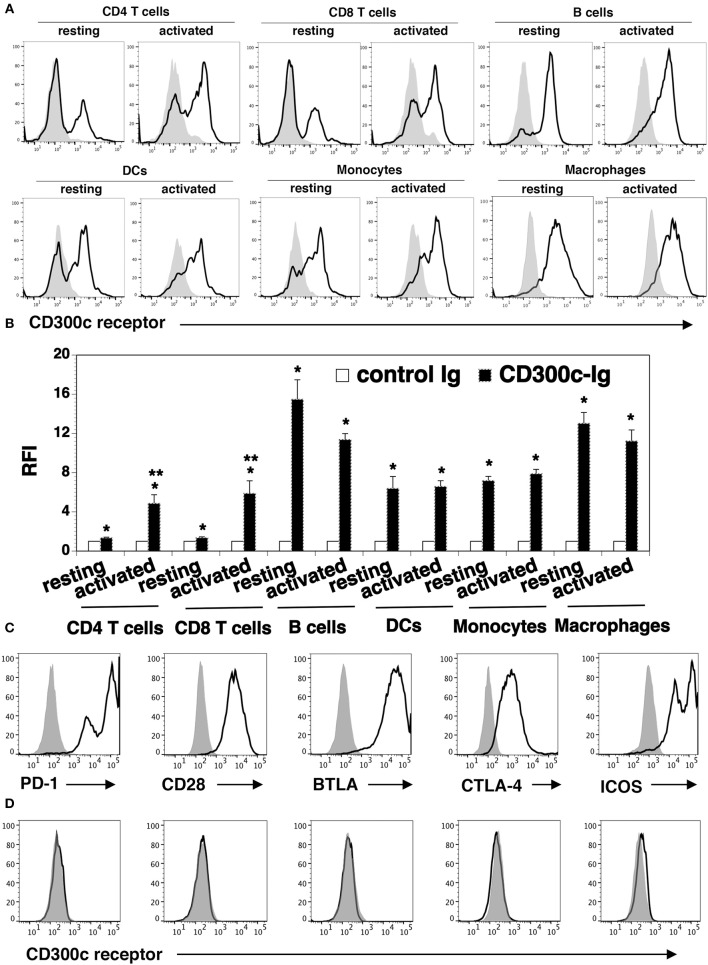
The expression pattern of the putative mCD300c2 counter-receptor. **(A,B)** Murine resting and activated immune cells were harvested or generated as in Figure 5A. The cells were stained with biotinylated mCD300c2-Ig (open histograms) or control Ig (shaded histograms), followed by streptavidin-PE. The binding of mCD300c2 to its putative receptor on immune cells was determined by flow cytometry. **(A)** Representative flow cytometric profiles and **(B)** statistical analysis showing the binding of mCD300c2-Ig or control Ig to resting and activated immune cells. **(B)** Data are presented as relative fluorescence intensity (RFI) for cell binding of mCD300c2-Ig vs. control Ig. The data were pooled from 3 independent experiments. **(A,B)** *P < 0.05 compared with control Ig. **P < 0.05 compared with resting cells. **(C,D)** HEK-293 cells were transfected with an expression vector containing the mouse CD28, CTLA-4, PD-1, BTLA, or ICOS gene. The transfected cells were stained with **(C)** antibodies against the respective CD28, CTLA-4, PD-1, BTLA, or ICOS protein (open histograms) or isotype Ab (shaded histograms), or **(D)** biotinylated mCD300c2-Ig (open histograms) or control Ig protein (shaded histograms), and analyzed by flow cytometry. Representative flow cytometric profiles showing the binding of **(A,D)** mCD300c-Ig or control Ig, or **(C)** indicated antibodies to **(A)** resting and activated immune cells, or **(C,D)** transfected HEK-293 cells.

To determine the expression pattern of the CD300c counter-receptor, purified mCD300c2-Ig and control Ig proteins were biotinylated. Splenocytes from C57BL/c mice were stained with the biotinylated proteins, followed by streptavidin-PE. Flow cytometric analysis showed that mCD300c2-Ig bound to resting CD4^+^ and CD8^+^ T cells, and the binding was increased when CD4^+^ and CD8^+^ T cells were activated by anti-CD3 and anti-CD28 antibodies (Figures 6A,B).

We also analyzed the expression of the CD300c counter-receptor on other immune cells. We found that mCD300c2-Ig bound to both resting and activated B220^+^ B cells, CD11c^+^ DCs, CD11b^+^ monocytes and G4/80^+^ macrophages (Figures 6A,B). The expression levels of the putative mCD300c counter-receptor on these immune cells were not significantly changed upon activation by LPS.

To determine whether mCD300c binds to molecules previously identified as receptors of the known B7 family members, HEK-293 cells were transfected with an expression vector containing the mouse CD28, CTLA-4, PD-1, BTLA, or ICOS gene. The expression of these receptors on the transfected 293 cells was confirmed by flow cytometric analysis with the antibodies against the respective receptors (Figure 6C). The binding of mCD300c to the transfected HEK-293 cells was then analyzed. As shown in Figure 6D, mCD300c2 did not bind to the CD28, CTLA-4, PD-1, BTLA, or ICOS transfected cells.

Taken together, our results suggest that the mCD300c2 counter-receptor is expressed on resting and activated CD4 and CD8 T cells, B cells, DCs, monocytes, and macrophages. The expression levels of the receptor on activated CD4 and CD8 T cells is upregulated. The mCD300c counter-receptor seems to be distinct from CD28, PD-1, CTLA-4, PD-1, or BTLA.

### hCD300c-Ig protein ameliorates GVHD in mice

**Figure 7:**
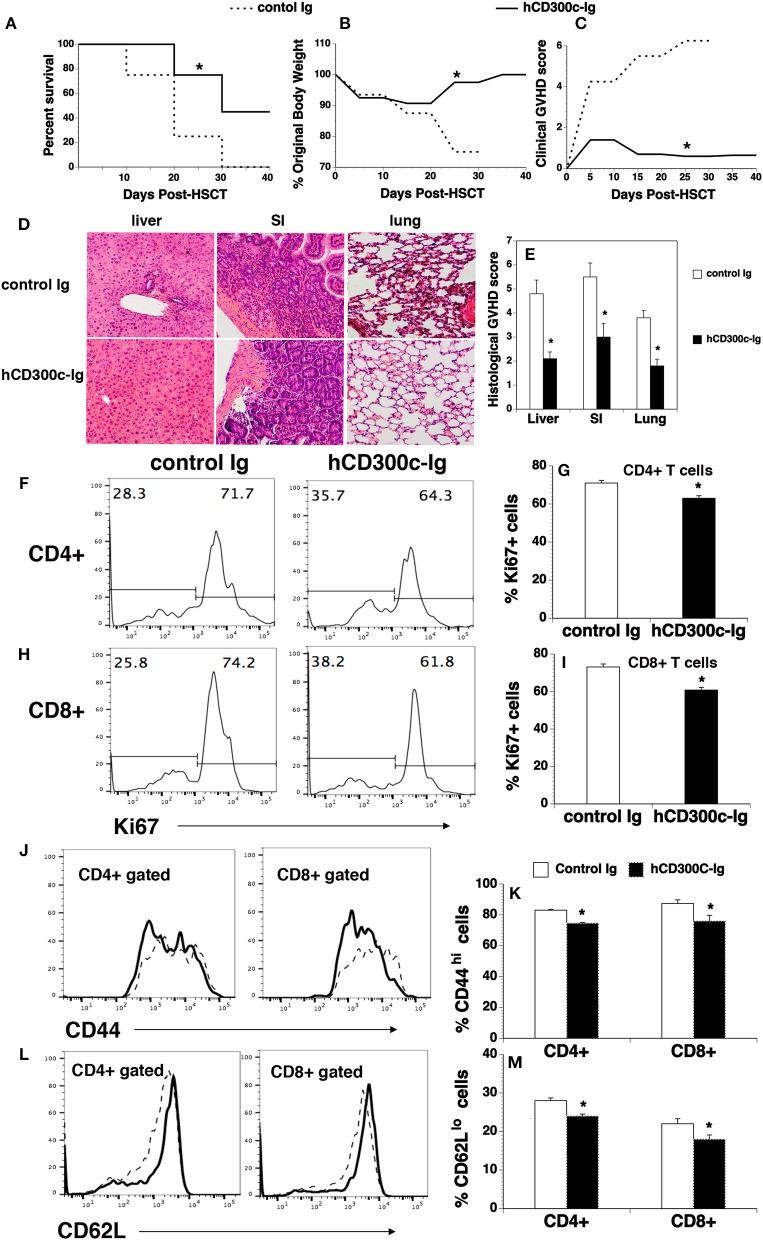
hCD300c-Ig ameliorates GVHD in mice. Lethally irradiated BALB/c recipients were injected i.v. with 5 × 10^6^ BM and 2.5 × 10^6^ spleen cells from C57BL/6 mice at day 0 and i.p. with 20 μg hCD300c-Ig, or control Ig every 3 days for 6 times. **(A–C)** Recipients were monitored for **(A)** survival, **(B)** weight change, and **(C)** clinical GVHD. **(D,E)** In separate experiments, recipients given 20 μg hCD300c-Ig or control Ig at 3-day intervals from days 0 to 12 were euthanized 2 weeks after BMT. **(D,E)** The liver, SI and lung were analyzed for histologic damage. **(D)** Representative photomicrographs (the magnification was X200), and **(E)** mean ± SD of histopathology scores. **(F–M)** hCD300c-Ig inhibits T-cell proliferation and activation in response to alloantigens *in vivo*. Lethally irradiated BALB/c mice were injected i.v. with 5 × 10^6^ BM 10 × 10^6^ splenic cells from C57BL/6 mice. The recipients were injected i.v. on day 0 and i.p. on day 2 with 20 μg hCD300c-Ig, or control Ig. On Day 4 post-transplant, the percentage of **(F–I)** Ki67^+^, **(J,K)** CD44^hi^, and **(L,M)** CD62L^lo^ cells in donor T cells (H2^b+^CD4^+^, or H2^b+^CD8^+^) of the spleens were examined by flow cytometry. **(F,H,J,L)** Representative flow cytometric profiles and **(G,I,K,M)** statistical data are shown. **(J,L)** Dash lines: control Ig; solid lines: hCD300c. Pooled data from 2 separate experiments are represented; with 5–6 mice per group in each experiment. *P < 0.05 compared with control Ig-treated mice.

Although bone marrow (BM) transplantation (BMT) has been widely used in the treatment of many diseases, GVHD remains a major complication after allogeneic BMT. Acute GVDH is primarily caused by T cells in donor transplants attacking recipient's tissues. We used a well-defined MHC-mismatched [C57BL/6 (H2^b^) → BALB/c (H2^d^)] GVHD mouse model to validate the effect of CD300c on T cells *in vivo*. BALB/c mice were lethally irradiated and injected i.v. with BM and splenic cells from allogeneic C57BL/6 mice. The recipients were then injected i.p. with hCD300c-Ig, or control Ig. The development of GVHD was monitored over time. As shown in Figures 7A–C, control Ig-treated GVHD recipients revealed gradual body weight loss and all succumbed by day 35 after BMT. hCD300c-Ig treatment significantly reduced the mortality and morbidity of GVHD, with 45% of the mice still surviving at day 40 post-transplantation (Figures 7A–C). GVHD severity was confirmed by pathologic analysis, showing that pathology scores of the liver, small intestine (SI), and lung in hCD300c-Ig-treated recipients were significantly lower than those in control Ig-treated recipients (Figures 7D,E). These data suggest that hCD300c-Ig treatment attenuates GVHD *in vivo*.

We then analyzed T cell proliferation, survival, and activation in hCD300c-Ig- or control Ig-treated GVHD mice. Lethally irradiated BALB/c recipients were injected i.v. with BM and splenic cells from C57BL/6 mice. The mice were injected i.v. on day 0 and i.p. on day 2 with 20 μg hCD300c-Ig or control Ig protein. The recipients were euthanized and the spleens were harvested on day 4. We analyzed for the expression of Ki67, a cell marker of proliferation. As shown in Figures 7F–I, the percentages of Ki67^+^ cells in donor CD4^+^ and CD8^+^ T cells of hCD300c-Ig-treated recipients were significantly lower than those in control Ig-treated mice. We also analyzed the survival of donor CD4^+^ and CD8^+^ T cells, and found that the percentages of annexin V^+^ 7-ADD^−^ apoptotic CD4^+^ or CD8^+^ T cells were not significantly different between hCD300c-Ig- and control Ig-treated groups (data not shown). We next examined the expression of activation markers by CD4^+^ and CD8^+^ T cells. Although the percentages of CD69^+^ cells in donor CD4^+^ and CD8^+^ T cells were not significantly different between hCD300c-Ig- and control Ig-treated groups (data not shown), the percentages of CD44^hi^ cells and CD62L^lo^ cells in donor CD4^+^ and CD8^+^ T cells were significantly reduced in hCD300c-Ig-treated GVHD mice (Figures 7J–M).

Taken together, our data suggest that hCD300c-Ig treatment attenuates GVHD, likely by inhibition of the proliferation and activation of donor T cells in response to alloantigen stimulation.

## Discussion

In an effort to identify additional immune regulators, the present study describes CD300c as a novel T cell co-inhibitory molecule. CD300c has a significant amino acid sequence and structural homology with the known B7 family members. CD300c protein is expressed on APCs and its counter-receptor is expressed on T cells. Functionally, CD300c-Ig protein inhibits the proliferation, activation and cytokine production of T cells. Therefore, CD300c contains typical features of B7 family members, suggesting that it is a B7 family-related molecule.

The nomenclature for mCD300c is still confusing (21). mCD300c is also called CLM-2, and mCD300c2 is also known as LMIR2/DIgR1/MAIR-II/CLM-4/CD300d (21, 22, 38–40). It has been reported that the mCD300c2 gene is located on mouse chromosome 11 (22, 38) and hCD300c is located near to human chromosome 17 (18), the syntenic region of mouse chromosome 11. The data further suggest that mCD300c2 is the mouse homolog of hCD300c (22, 38).

The B7 family is a member of the Ig superfamily. The extracellular region of the known B7 family members typically contain IgV and IgC domains. However, both human and mouse CD300c have only one IgV domain in the extracellular region. It has been reported that the interaction site in the Ig superfamily members is often mapped to the distal Ig domain, which would be the IgV domain in the CD300c. Therefore, the absence of the IgC domain in the CD300c would not significantly reduce its ability to inhibit T cell functions.

We have shown that the mCD300c2 protein is expressed on the cell surface of a variety of APCs, including B cells, monocytes, macrophages and DCs. It has been reported that that mCD300c2 mRNA was abundant in the spleen (21, 22, 38, 39) and that mCD300c2 protein was expressed on cell surface of DCs, monocytes, macrophages and B cells (21, 22, 38, 39). High hCD300c transcript levels have also been detected in the human spleen and thymus (28) and hCD300c protein has been shown on human monocytes, macrophages, granulocytes, and DCs, as well as, in a subpopulation of B and T cells (19, 24, 28). Our results are consistent with these reports.

Our results demonstrate that the mCD300c3 counter-receptor is expressed on resting and activated CD4 and CD8 T cells, B cells, DCs, monocytes, and macrophages. The expression levels of the counter-receptor on activated CD4 and CD8 T cells is upregulated upon activation, while the expression levels of the mCD300c counter-receptor on resting and activated B cells, DCs, monocytes, and macrophages were not significantly different. mCD300c2 protein did not bind to CD28, PD-1, CTLA-4, PD-1, or BTLA-expressing cells, indicating that the mCD300c2 counter-receptor is distinct from known members of the CD28 receptor family. CD300c is also considered as a receptor, and lipids such as phosphatidylserine and phosphatidylethanolamine can act as ligands for CD300c (23, 31, 41). It remains to be determined whether hCD300c and mCD300c2-Ig affects T cell functions through these lipids or a protein counter-receptor.

The expression of CD300c protein on APCs and its counter-receptor on T cells suggests that CD300c affects T cells. Indeed, we have demonstrated that both hCD300c and mCD300c2 significantly inhibit the proliferation, activation, and/or cytokine production of CD4 and CD8 T cells *in vitro*. It seems that the inhibition of the proliferation of murine T cells by hCD300c-Ig was slightly greater than that of human T cells (Figure 2A vs. Figure 2K), whereas the inhibition of the expression of CD69 on human T cells was greater than that on murine T cells (Figures 2H,J vs. Figures 2L,M). The phenomena may be related to the expression of the CD300c counter-receptor on T cells because we found that murine and human CD4 and CD8 T cells expressed different levels of CD300c counter-receptor (Figure 6B vs. Supplemental Figure 3).

We have also shown that hCD300c-Ig treatment attenuates acute GVHD in mice. To the best of our knowledge, this is the first report that CD300c is able to inhibit T cell function and treat GVHD. The effect of CD300c on GVHD is associated with the inhibition of T cell function *in vivo*. In agreement with the *in vitro* data, hCD300c-Ig inhibits T cell proliferation and activation in the GVHD model. However, although both mCD300c2-Ig and hCD300c-Ig inhibit the expression of CD69 in T cells *in vitro*, we did not observe that hCD300c-Ig treatment reduced CD69 expression by donor T cells *in vivo*. This inconsistency is most likely caused by time differences in analyzing this marker. CD69 is an early activation marker. We analyzed the expression of this marker 1 day after activation by anti-CD3 antibody or anti-CD3 and anti-CD28 antibodies *in vitro*, but 4 days after activation by allogeneic antigens in the GVHD model. hCD300c-Ig may inhibit the expression of CD69 *in vivo* at early time points, but this inhibition was not in effect 4 days later. This notion is supported by our results that hCD300c-Ig reduced the percentages of two other T cell activation markers CD44^hi^ cells and CD62L^lo^ cells, in CD4^+^ and CD8^+^ T cells *in vitro* 3 days (Figures 3M–Q) and *in vivo* 4 days (Figures 7J–M) after activation.

It has been reported that CD300c has effects on other types of immune cells (21–30). Crosslinking of CD300c on NK cells by its antibody induce cytokine secretion and degranulation (23). CD300c is also involved in the modulation of IgE-mediated basophil activation (30). mCD300c2 non-covalently associates with the signaling adaptor DAP10 in macrophages and B cells (27, 34). mCD300c negatively regulates adaptive immune responses by B cells in a DAP12-dependent manner. mCD300c2–deficient B cells had an enhanced proliferation in response to BCR and CpG stimulation. In contrast, expression of mCD300c2 into mCD300c2^−/−^ B cells was able to suppress BCR- and CpG-mediated proliferation (24, 35). In addition, mice deficient in MAIR-II (LMIR-2) are more susceptible to caecal ligation and puncture-induced peritonitis than wild-type mice (36). Furthermore, cross-linking of hCD300c by its antibody on DCs resulted in decreased expression of MHC-II and reduced production of TNFα and IL-6, but increased production of type I of IFN (29, 37). In contrast, mCD300c2 stimulates proinflammatory cytokines and chemokine secretions from macrophages. Cross-linking of mCD300c2 on macrophages resulted in increased production of TNFα, IL-6, and MCP-1 (27). It is unclear whether the effects of CD300c on these immune cells are caused by a direct effect. Similarly, although we used purified T cells for the [^3^H] thymidine incorporation and cytokine production assays, we used splenocytes that include T cells and other immune cells for the T cell activation studies. The possibility that the inhibition of T cell activation by CD300c-Ig may be caused by its indirect effect on other immune cells cannot be excluded at this stage.

In summary, we have identified CD300c as a B7 family-related molecule. CD300c protein is expressed on APCs, and its counter-receptor is expressed on T cells. Soluble human or mouse CD300c-Ig fusion proteins significantly inhibit T cell proliferation, activation, and cytokine production *in vitro*. Administration of hCD300c-Ig protein attenuates GVHD in mice. Therefore, CD300c protein has the potential to be used in the treatment of GVHD, autoimmune disease, and transplant rejection.

## Author contributions

CC performed experiments and analyzed data. MS and YL performed experiments. LL designed experiments, analyzed data, supervised the study, and wrote the manuscript.

### Conflict of interest statement

The authors declare that the research was conducted in the absence of any commercial or financial relationships that could be construed as a potential conflict of interest.

## Abbreviations

APC: antigen-presenting cells
DCs: dendritic cells
GVHD: graft-vs. -host disease
ICOSL: T cell co-stimulator ligand
Ig: immunoglobulin
CD300c-Ig: CD300c-IgG2a Fc
CFSE: carboxyfluorescein diacetate succinimidyl ester
hCD300c: human CD300c
mCD300c: mouse CD300c
CLM-6: CMRF-35-like molecule-6
LMIR2: leukocyte mono-Ig-like receptor 2
DIgR1: dendritic cell-derived Ig-like receptor 1
MAIR-II: myeloid-associated Ig-like receptor II
MW: molecular weight
BM: bone marrow
BMT: BM transplantation
SI: small intestine.
