# Supplementary material for: A CD300c-Fc Fusion Protein Inhibits T Cell Immunity
Source: Front Immunol. 2018 Nov 15;9:2657. doi: 10.3389/fimmu.2018.02657 (PMC6249344; doi:10.3389/fimmu.2018.02657)
Supplement: Supplementary file 1 [file Data_Sheet_1.pdf]

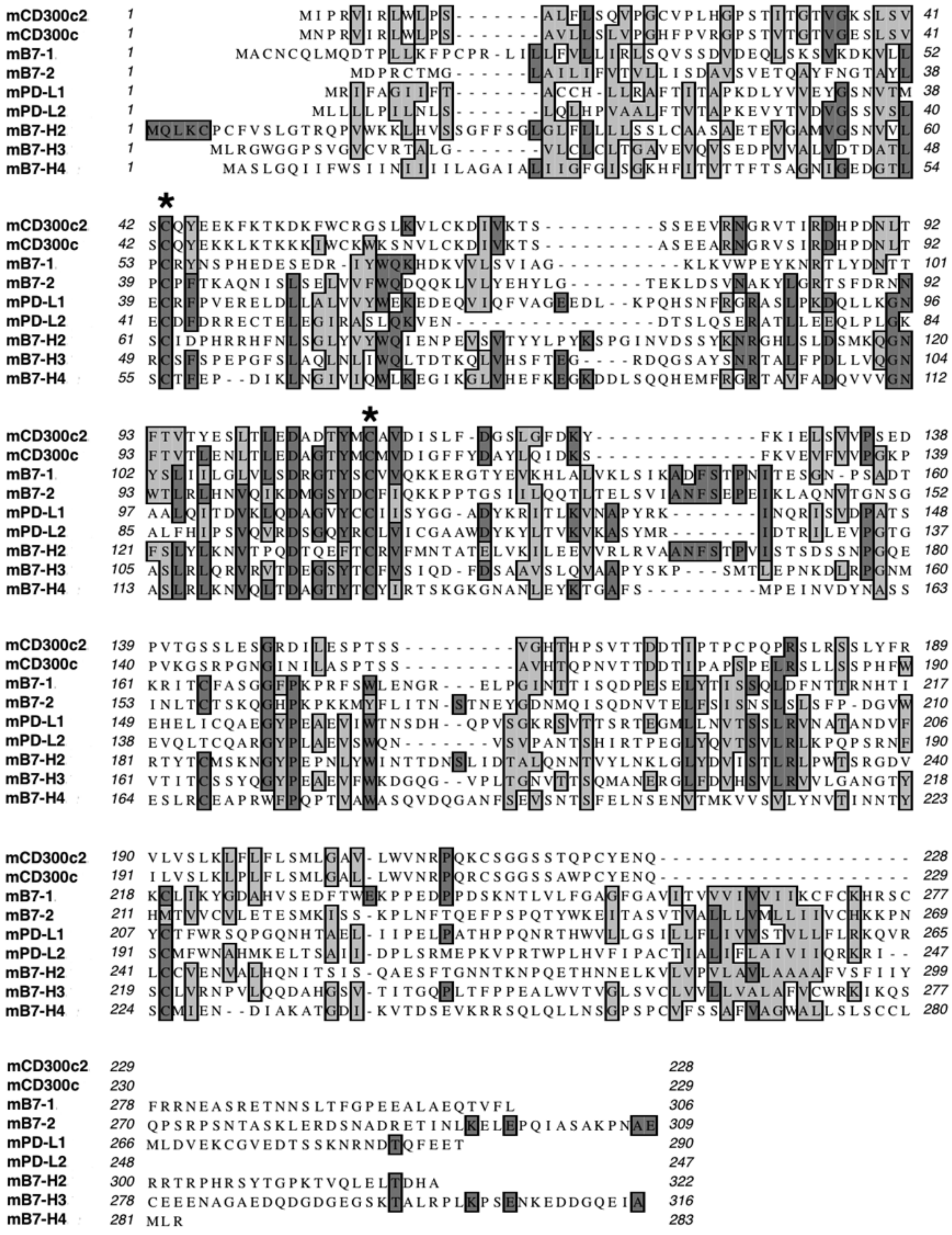

Supplemental Figure 1. Alignment of mCD300c2 and mCD300c with some known murine B7 family members. Identical amino acids are shaded black. Amino acids with strong homologies are shaded in gray. Conserved cysteine residues are labelled with an asterisk (*).

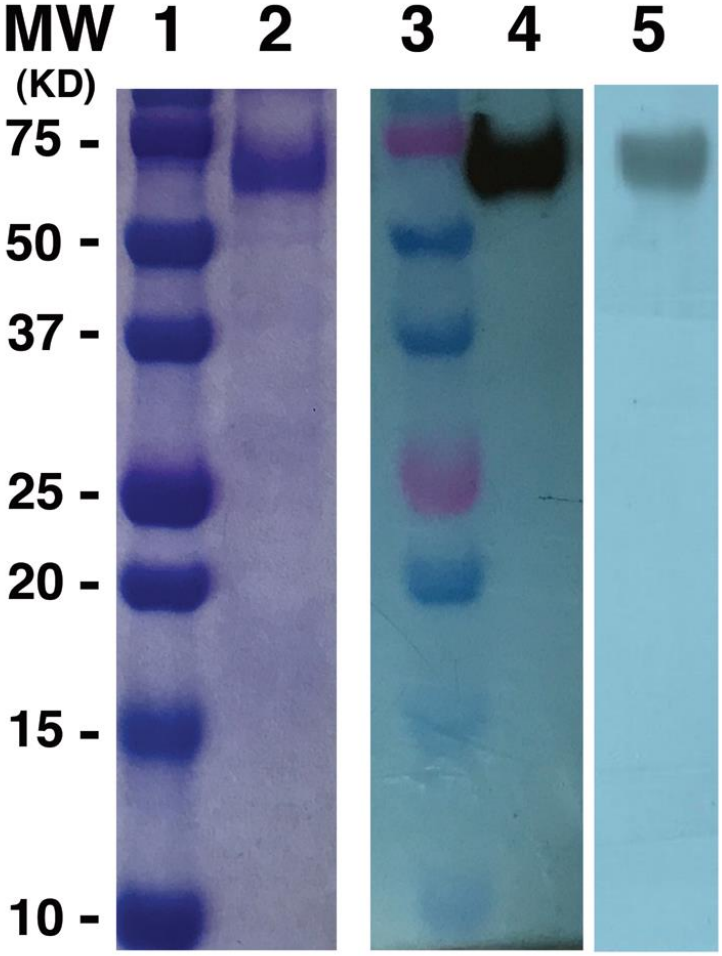

Supplemental Figure 2. Characterization of purified hCD300c-Ig protein. Gel and blot show purified hCD300c-Ig protein; Lanes 1 and 3: MW markers; lane 2: Coomassie blue-stained SDS-PAGE; lane 4: Western blot with anti-IgG2a antibody; lane 5: Western blot with anti-hCD300c antibody

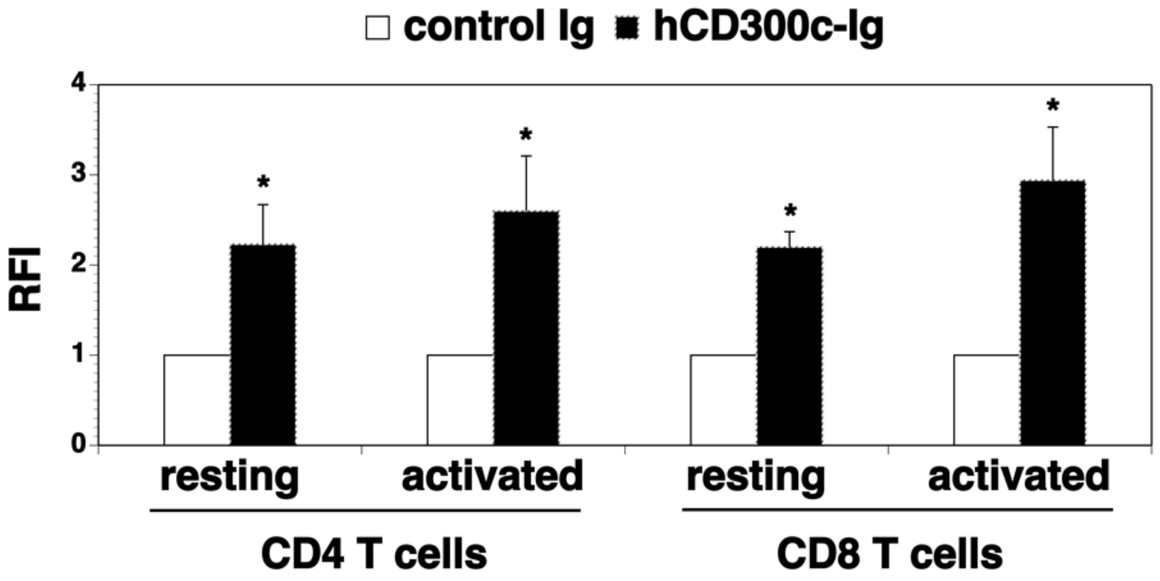

Supplemental Figure 3. The expression pattern of the putative hCD300c counter-receptor on human T cells. Human peripheral blood mononuclear cells (obtained from AllCells, LLC.) were cultured with anti-human CD3 and CD28 antibodies for 3 days. Resting and activated cells were stained with biotinylated hCD300c-Ig or control Ig, followed by streptavidin-PE. The binding of hCD300c to its putative receptor on CD4 and CD8 T cells was determined by flow cytometry. The data were pooled from 3 independent experiments and presented as relative fluorescence intensity (RFI) for cell binding of hCD300c-Ig versus control Ig. * $P<0.05$ compared with control Ig.
